# A sequencing study of *CTLA4* in Pakistani rheumatoid arthritis cases

**DOI:** 10.1371/journal.pone.0239426

**Published:** 2020-09-18

**Authors:** Muhammad Muaaz Aslam, Fazal Jalil, Peter John, Kang-Hsien Fan, Attya Bhatti, Eleanor Feingold, F. Yesim Demirci, M. Ilyas Kamboh

**Affiliations:** 1 Atta-ur-Rahman School of Applied Biosciences, National University of Sciences and Technology, Islamabad, Pakistan; 2 Department of Human Genetics, Graduate School of Public Health, University of Pittsburgh, Pittsburgh, PA, United States of America; 3 Department of Biotechnology, Abdul Wali Khan University, Mardan, Pakistan; Soroka University Medical Center, ISRAEL

## Abstract

Rheumatoid arthritis (RA) is a multifactorial autoimmune disease. The interaction of genetic and environmental factors is likely necessary for RA. Among potential genetic factors, many major histocompatibility complex (MHC) and non-MHC variants may be involved in RA susceptibility. *CTLA4* is involved in the regulation of T-cell response during an immune reaction, and multiple *CTLA4* single nucleotide polymorphisms (SNPs) have been associated with numerous autoimmune diseases, including RA. To our knowledge, the genetic association of *CTLA4* with RA risk has not been examined previously in the Pakistani population. In this study, we sequenced the entire *CTLA4* gene and flanking regions in 95 Pakistani RA cases followed the screening of identified variants in Study 1 sample consisting of 350 RA cases and controls. Four common significant variants identified in Study 1 sample were further examined in a larger Study 2 replication sample comprising 1,678 independent RA cases and controls. We report significant associations of three variants from the combined analysis: rs3087243 (OR = 1.26, p = 4.47E-03), rs5742909 (OR = 1.78, p = 4.60E-03), and rs11571319 (OR = 1.48, p = 6.64E-03); the latter is a novel association in the Pakistani sample.

## Introduction

RA is an inflammatory, chronic, autoimmune syndrome that causes articular damage, synovial joint destruction, and related comorbidities [1]. RA is characterized by increased levels of autoantibodies, inflamed bones and joints, synovitis, and destruction of bone and cartilage that lead to fatigue, chronic pain, and in the worst cases, permanent disability [2]. A complex network of immune cells (B-cells, T-cells, mast cells, plasma cells, and dendritic cells) and cytokines (pro-inflammatory and anti-inflammatory) are involved in the etiology of RA [3]. Globally, RA affects almost 0.5 to 1% of the general population [4]; in Pakistan, its prevalence is approximately 0.5% [5]. RA can affect both sexes at any age, but the prevalence of RA is higher in women than men [6]. The interplay of genetic and environmental factors (climate, diet, geography, smoking, and microbiome) leads to the onset of RA [7, 8]. Class II major histocompatibility complex (MHC-II) is the most important genetic locus for RA susceptibility. Many genome-wide association studies (GWAS) and meta-analyses of GWAS have identified more than 100 single nucleotide polymorphisms (SNPs) loci associated with RA susceptibility. Most of the identified single-nucleotide polymorphisms (SNPs) in these loci are clustered around immune-related genes [9].

Cytotoxic T-lymphocyte antigen-4 (*CTLA4*) also known as *CD152*, is an inhibitory glycoprotein present on the surface of T-cells. It regulates the activation of autoreactive T-cells, tolerance against self-antigens, and inhibits the differentiation of monocytes into osteoclasts [10]. T-cells have a key role in RA derived autoimmune response, therefore mediator of T-cells such as *CTLA4* has a regulatory role in RA pathogenesis [11]. The human *CTLA4* gene is present on the long arm of chromosome 2 at position 33.2q. It belongs to the immunoglobulin superfamily and consists of four exons [12]. Exon one contains the sequence for extracellular leader peptide, exon two encodes extracellular ligand-binding site, exon three encodes the transmembrane domain and exon four encodes the cytoplasmic tail [13]. Several genetic studies have reported the association of *CTLA4* SNPs with numerous autoimmune conditions, including RA [14]. *CTLA4*/rs231775 (+49A/G), rs3087243(CT60 G/A) and rs5742909 (-318 C/T) are most widely studied for their associations with RA susceptibility in different populations [15, 16].

Hitherto, most of the genetic studies on RA have been conducted on East-Asians, Europeans, or European-derived populations, with limited genetic data available in the Pakistani population. In an effort to comprehensively examine the role of *CTLA4* genetic variation with RA susceptibility in Pakistanis, we re-sequenced the entire *CTLA4* gene in 95 RA cases and then examined the identified variants in more than 2,000 cases and controls.

## Materials and methods

### Subjects

A total of 2,028 subjects comprising 1,291 RA cases (mean age ± SD = 41.8 ± 12.36, 76.2% women), and 737 controls (mean age ± SD = 40.7 ± 12.49, 39.5% women) were derived from our two published studies [2, 17]. While Study 1 comprised 239 cases and 111 controls collected from October 2009 to December 2012 and Study 2 consisted of 1,222 cases and 737 controls collected from September 2015 to May 2017. After obtaining Institutional Review Board (IRB) approvals, blood samples were collected from five rheumatology facilities located in two adjacent cities in Pakistan: Pakistan Institute of Medical Sciences, Military Hospital, Fauji Foundation Hospital, and Rehmat Noor Clinic in Islamabad, and Kahota Research Laboratories Hospital in Rawalpindi. All recruited RA cases were diagnosed by rheumatologists following the 1987 ACR (American College of Rheumatology) classification criteria [18]. All control subjects were recruited from the general population and had no history of any autoimmune disease at the time of enrollment. Written informed consent was taken from all study participants at the time of recruitment. Blood samples were collected in EDTA coated tubes to avoid coagulation and processed shortly after the collection. This study was approved by the IRB of the University of Pittsburgh, USA (IRB no. PRO12110472).

### Genomic DNA extraction

Standard phenol-chloroform extraction method or GeneJET Whole Blood Genomic DNA Purification (Thermo Scientific USA) was used to extract genomic DNA from whole blood and NanoDrop^TM^ 2000 spectrophotometer (Thermo Scientific USA) was used for quantification.

### *CTLA4* sequencing

The entire *CTLA4* gene (all four exons and introns) and flanking regions on chromosome 2 (hg19, chr2: 203866788–203874960) were polymerase chain reaction (PCR)-amplified using nine sets of primers in 95 RA-cases from Study 1. PCR primer sequences are available in **S1 Table**. All primers were designed using Primer3 software (http://frodo.wi.mit.edu/primer3/). Automated DNA sequencing of PCR products was performed in a commercial lab (Beckman Coulter Genomics, Danvers, MA) using the Sanger method on ABI 3730×l DNA Analyzers. The sequences were aligned against a reference sequence (NM_005214) by Variant Reporter™ Software v1.0 (Thermo Scientific USA) to identify variants.

### Genotyping

Follow-up genotyping of sequence variants was performed on 350 subjects from Study 1 using iPLEX^®^ Gold (Sequenom). Genotype call rate ≥98%, concordance with Hardy-Weinberg Equilibrium (HWE), and discrepancy rate <1% were used as QC measures. The iPLEX^®^ Gold (Sequenom) genotyping was performed in the Core Laboratories of the University of Pittsburgh, Pittsburgh, USA. Follow-up genotyping of 4 selected SNPs from Study 1 was conducted on Study 2 samples (1,052 RA cases, 626 healthy controls) using TaqMan® (Applied Biosystems, ThermoFisher Scientific) genotyping assays (C___2415786_20, C__30981401_10, C__30981401_10 and C___3296043_10) following manufacturer’s guidelines. The 384-wells plates containing dried DNA were used in both genotyping methods. After thermal cycling of functionally tested TaqMan® assay, QuantStudio™ 12K Flex system (Applied Biosystems, ThermoFisher Scientific) was used for the end-point fluorescence reading of 384-wells DNA plates. Sequences of iPLEX^®^ Gold (Sequenom) genotyping primers are available upon request.

### Statistical analysis

Haploview 4.0 [19] (www.broadinstitute.org/haploview) was used to analyze variants identified through sequencing for their minor allele frequency (MAF) and linkage disequilibrium (LD) patterns. A chi-square goodness of fit test was used to check the concordance with Hardy-Weinberg Equilibrium (HWE). Logistic regression was employed for case-control association analysis using sex and age as covariates. Association of significant variants with anti-cyclic citrullinated peptide (anti-CCP) and rheumatoid factor (RF) seropositivity was also examined using logistic regression with age and sex as covariates. p<0.05 was considered as suggestive evidence of association. All association analyses were implemented in R version 3.4.4 (http://www.r-project.org).

## Results

### *CTLA4* sequencing

Sequencing of the entire *CTLA4* gene and flanking regions in 95 Pakistani RA patients identified 30 variants, including two novel variants (GRCh38: 203869988 and GRCh38: 203870218). Most of the variants were in intronic regions and only one coding variant (rs231775) was identified. Four variants (rs231774, rs231773, rs11571317, and rs5742909) were in the 5’ upstream region, one was in 3’UTR (rs11331867) and three (rs231721, rs11571319, and rs3087243) were in the downstream region.

### Genotyping of *CTLA4* variants in Study 1 sample

**Fig 1:**
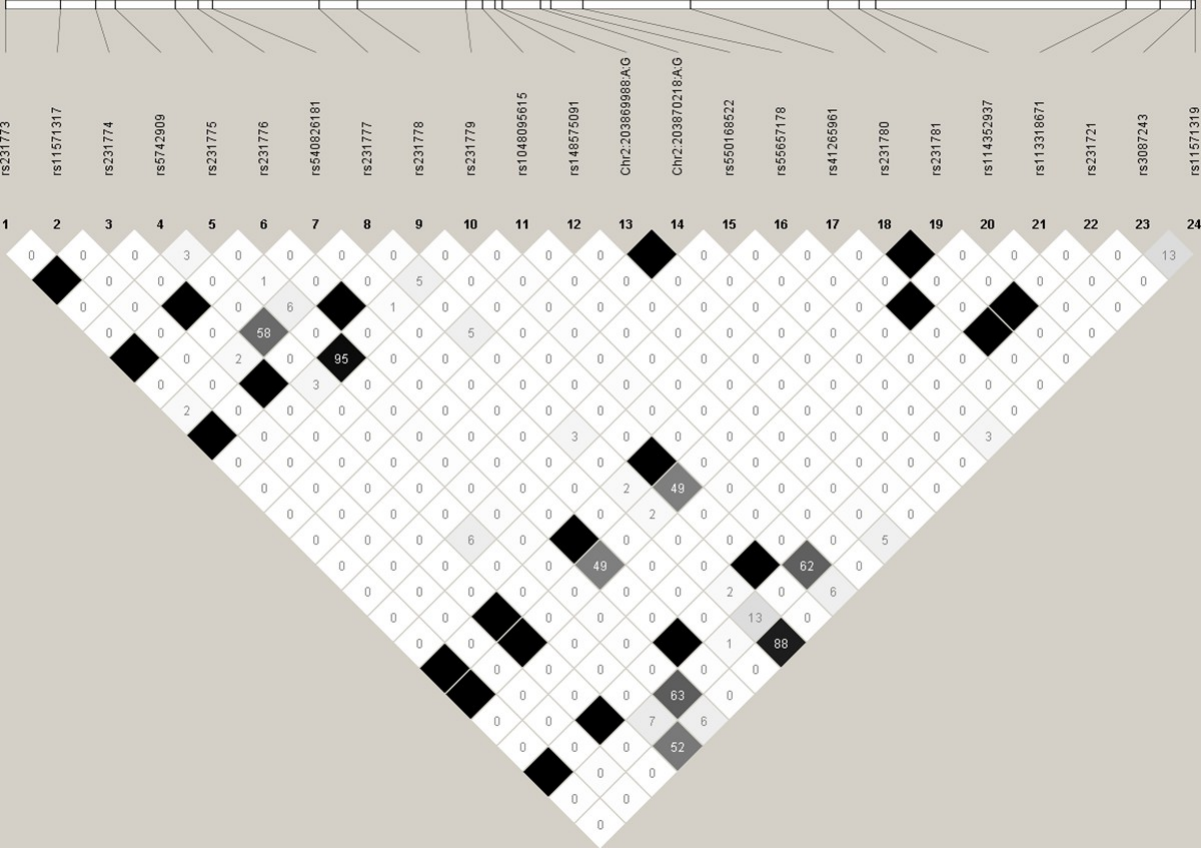
LD plot of genotyped 24 SNPs.

**Fig 2:**
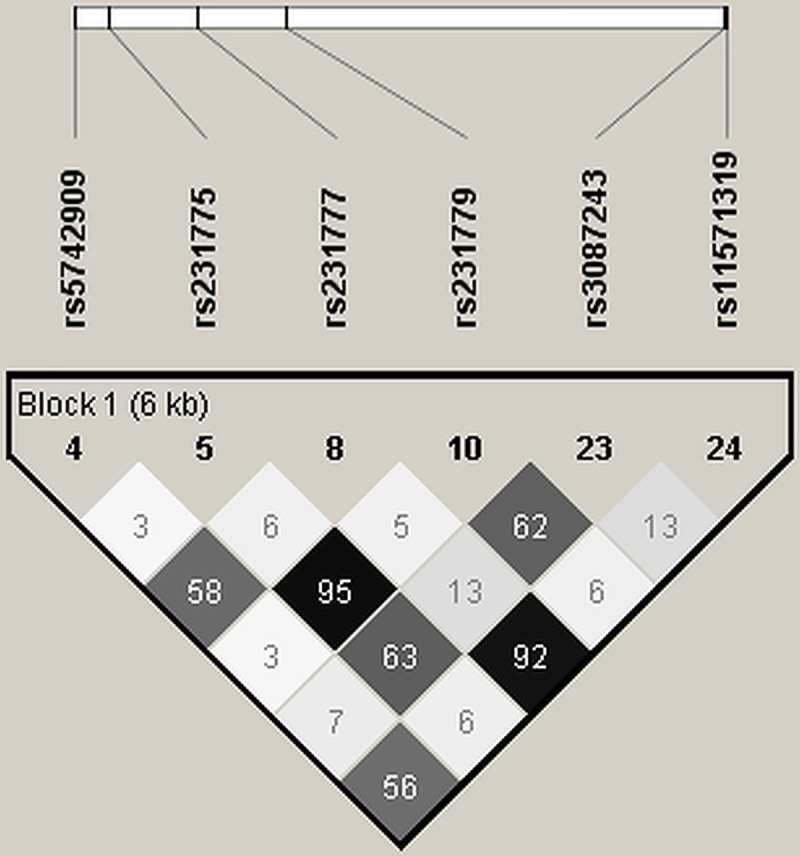
LD plot of 6 common SNPs.

Of 30 sequence variants identified in the discovery phase, 24 were successfully confirmed/genotyped in 350 individuals. The genotype call rate for all SNPs was ≥98% and they were in concordance with HWE. Out of 24 variants, 12 were rare (MAF <1%), 6 were uncommon (MAF between 1 to 5%) and 6 were common (MAF >5%) **(Table 1, Fig 1).** Among those only observed in cases, eight variants (rs231781, GRCh38: 203869988, GRCh38: 203870218, rs550168522, rs231774, rs231780, rs231721 and rs231773) were singleton, two variants (rs231776 and rs231778) were observed in two individuals. We did not observe any rare or less common variant in more than two independent cases. Case and control allele frequencies were similar for most variants with >1% MAF in the Study 1 sample, except for six most common SNPs (rs231775, rs231779, rs231777, rs11571319, rs5742909, and rs3087243) that showed a trend for the association (p-range = 0.2 to 0.8). There was a strong linkage disequilibrium (LD) between rs231775 and rs231779 (r^2^ = 0.95), and between rs231777 and rs11571319 (r^2^ = 0.92) (**Fig 2**).

**Table 1:** 24 SNPs identified by sequencing and successfully genotyped in extended samples.

SNP Name	SNP ID	Position	Variant Type	Minor Allele	MAF	p-value
CTL4p5377	rs231781	203872164	Intron	A	0.14	9.88E-01
CTL4p3201	GRCh38: 203869988	203869988	Intron	G	0.14	9.88E-01
CTL4p3495	rs550168522	203870282	Intron	T	0.14	9.88E-01
CTL4p713	rs231774	203867500	Upstream	T	0.14	9.88E-01
CTL4p5187	rs231780	203871974	Intron	G	0.14	9.88E-01
CTL4p7215	rs231721	203874002	Downstream	C	0.14	9.88E-01
CTL4p3431	GRCh38: 203870218	203870218	Intron	G	0.15	9.88E-01
CTL4p162	rs231773	203866949	Upstream	G	0.15	9.88E-01
CTL4p3691	rs55657178	203870478	Intron	T	0.29	9.82E-01
CTL4p1340	rs231776	203868127	Intron	A	0.29	9.82E-01
CTL4p2311	rs231778	203869098	Intron	G	0.29	9.82E-01
CTL4p3079	rs1048095615	203869866	Intron	A	0.29	5.97E-01
CTL4p5477	rs114352937	203872264	Intron	T	1.01	4.04E-01
CTL4p4351	rs41265961	203871138	Intron	A	1.01	3.98E-01
CTL4p1429	rs540826181	203868216	Intron	C	1.29	6.12E-01
CTL4p498	rs11571317	203867285	Upstream	T	1.15	3.52E-01
CTL4p7008	rs113318671	203873795	3’ UTR	T	1.44	5.34E-01
CTL4p3154	rs148575091	203869941	Intron	T	1.89	5.52E-01
CTL4p837	rs5742909	203867624	Upstream	T	5.76	5.30E-01
CTL4p2078	rs231777	203868865	Intron	T	9.97	8.63E-01
CTL4p7428	rs11571319	203874215	Downstream	A	10.93	7.67E-01
CTL4p1204	rs231775	203867991	Exon	G	32.41	2.00E-01
CTL4p2977	rs231779	203869764	Intron	T	32.46	2.05E-01
CTL4p7409	rs3087243	203874196	Downstream	G	43.93	4.07E-01

### Genotyping of selected *CTLA4* variants in Study 2 sample

Four of the six SNPs that showed the smallest p-values in the Study 1 samples were examined in the Study 2 sample. Because of the strong LD between rs231775 and rs231779 and between rs231777 and rs11571319), only one SNP from each pair (rs231775 and rs11571319) was selected for follow up genotyping. The genotype call rate in Study 2 sample was >90% for all four SNPs and they were in concordance with HWE. Statistically significant associations of rs3087243 (p = 4.47E-03, OR = 1.26), rs11571319 (p = 6.64E-03, OR = 1.48) and rs5742909 (p = 4.60E-03, OR = 1.78) were observed with RA risk **(Table 2).**

**Table 2:** Common *CTLA4* SNPs genotyped in 1,678 RA case-control subjects.

SNP	Position	Variant Type	Minor Allele	MAF	OR (95% CI)	p-value
rs231775	203867991	Exon	G	0.32	1.11 (0.94, 1.29)	2.21E-01
rs3087243	203874196	Downstream	G	0.43	1.26 (1.02, 1.38)	4.47E-03
rs11571319	203874215	Downstream	A	0.10	1.48 (1.11, 1.94)	6.64E-03
rs5742909	203867624	Upstream	T	0.05	1.78 (1.18, 2.60)	4.60E-03

OR = Odds ratio; CI = Confidence interval

### Association with anti-CCP and RF

Anti-CCP and RF data were available on 1,010 RA patients; of which, 877 were positive for anti-CCP and 914 for RF. In order to examine if RA-associated SNPs also affect seropositivity of anti-CCP or RF, we performed logistic regression analyses, but found no significant associations (**Table 3).**

**Table 3:** Association results of tested *CTLA4* SNPs with anti-CCP and RF seropositivity.

			Anti-CCP		RF	
SNP	Minor Allele	MAF	OR (95% CI)	p-value	OR (95% CI)	p-value
rs11571319	A	0.10	1.28 (0.71, 2.05)	0.37	0.95 (0.55, 1.52)	0.85
rs5742909	T	0.05	1.18 (0.57, 2.49)	0.66	0.97 (0.47, 2.05)	0.95
rs231775	G	0.31	0.96 (0.66, 1.21)	0.80	1.30 (0.90, 1.81)	0.18
rs3087243	G	0.44	1.00 (0.63, 1.12)	1.00	1.19 (0.74, 1.38)	0.33

OR = Odds ratio; CI = Confidence interval

## Discussion

**Fig 3:**
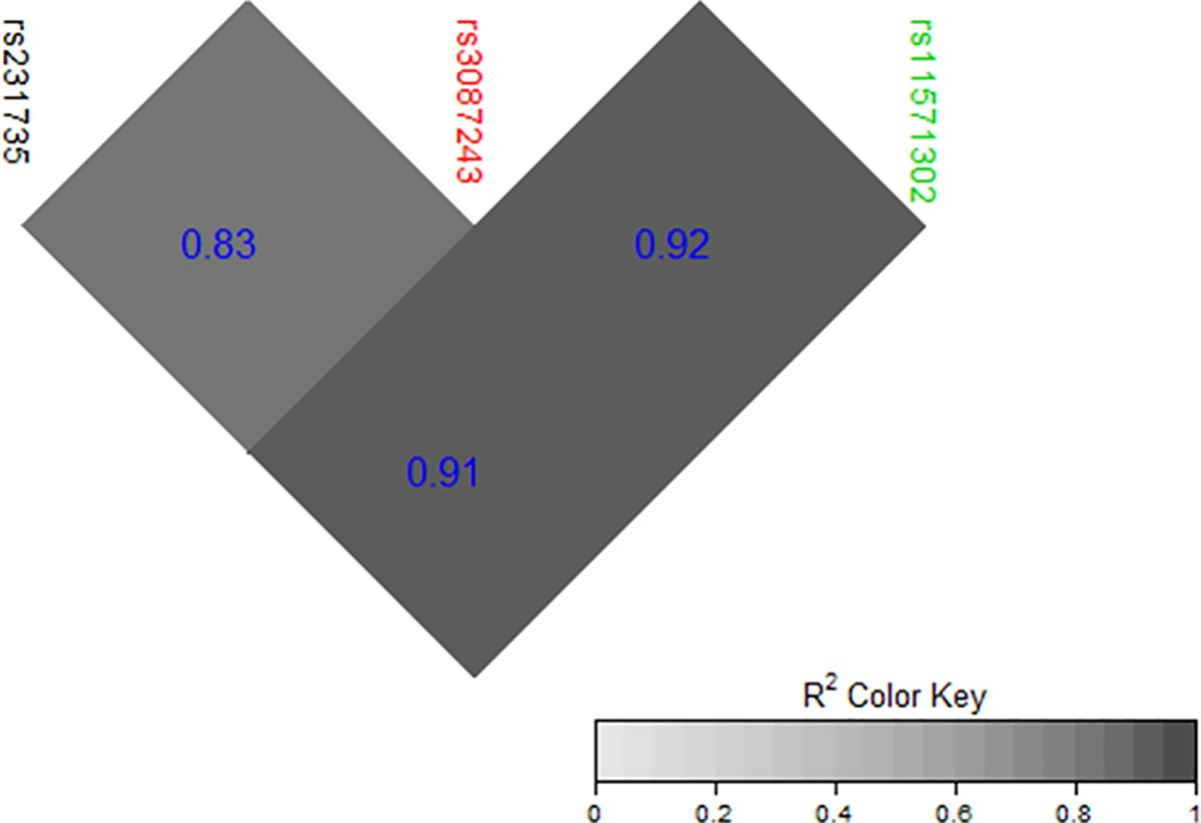
LD plot of previously published *CTLA4* SNPs and our tested common SNP.

Rheumatoid arthritis is a multifactorial, inflammatory autoimmune disease [20]. Many MHC and non-MHC genetic variants are associated with RA susceptibility [21]. *CTLA4* regulates the T-cell response in an immune reaction and genetic variation *CTLA4* has been reported to be associated with RA susceptibility [22]. To further explore the role of *CTLA4* genetic variation with RA susceptibility, we sequenced the entire *CTLA4* gene and flanking regions in Pakistani RA patients. A total of 30 variants were identified, including two novel ones; the latter were observed in singleton. Twenty four of the 30 variants were successfully genotyped in Study 1 sample of 350 RA cases and controls. Six of them (rs231775, rs231779, rs231777, rs11571319, rs5742909, and rs3087243) were selected for follow-up. After considering high LD between two sets of SNP pairs (rs231775 with rs231779, and rs231777 with rs11571319), four SNPs were moved forward in a larger replication sample of 1,678 RA cases and controls (Study 2 sample) where 3 showed statistically significant associations (rs3087243, rs11571319, rs5742909). Previously, rs3087243 has been reported to be associated with multiple diseases [23–25] and autoimmune conditions such as type 1 diabetes [26] and RA [27]. Similarly, rs5742909 has been reported to be associated with RA susceptibility in Canadians [15] and Egyptians [28]. In our study, rs3087243/G was associated with an increased risk of RA, as has also been reported in Europeans and Asians [27, 29]. However, in Mexicans rs3087243/G was associated with decreased RA risk [30], but it showed no association in the Polish population [31]. Two RA GWAS-implicated *CTLA4* SNPs (rs11571302, a downstream variant, and rs231735, an upstream variant), which were not covered in our sequencing sample, are also in LD with rs3087243 (**Fig 3**) [32, 33]. We observed a novel association of rs11571319 with RA in the Pakistani sample. To our knowledge, rs11571319 has not been reported previously to be associated with RA risk, although its association has been reported with Graves’ disease [34] and asthma [35]. Anti-CCP and RF seropositivity confer acute disease activity in RA patients [36] and may explain the observed genetic associations with RA risk. However, in this study, we could not establish this link, indicating that the association of *CTLA4* variants with RA risk is independent of anti-CCP and RF status, as has also been shown for other RA-associated genetic markers [37].

In rare cases, mutations in a single gene such as *MEFV* (heterozygous mutations in exon 2 and exon 3) can also lead to the onset of RA as a consequence [38]. In these circumstances, population-based preventive genomic sequencing (PGS) for the genomic screening can help to identify the genetic health risk in the general population [39].

To the best of our knowledge, this is the first study of the genetic association of *CTLA4* with the risk of RA in the Pakistani population where we found three significant associations, including one novel association. Our findings may have clinical implications with RA treatment outcome if confirmed in independent and larger studies; similar to those investigating the pharmacogenomics of drug therapy in RA [40, 41].

## Supporting information

S1 Table The sequence of *CTLA-4* primers in 5’ to 3’ direction. (DOCX)
