# Supplementary material for: A sequencing study of CTLA4 in Pakistani rheumatoid arthritis cases
Source: PLoS One. 2020 Sep 18;15(9):e0239426. doi: 10.1371/journal.pone.0239426 (PMC7500603; doi:10.1371/journal.pone.0239426)
Supplement: S1 Table — (DOCX) [file pone.0239426.s001.docx]

| **Forward Primer** | **Reverse Primer** | **Amplicon Length (base pairs)** |
| --- | --- | --- |
| 5′CTGTTTGCATGTCAGCCTTCTA3′ | 5′GGATCCTGAAGCTTTGAAATGT3′ | 1053 |
| 5′GGACCCTTGTACTCCAGGAA3′ | 5′GAAGGCATTCTTCCCACAAT3′ | 1000 |
| 5′TTGAGCTGGGTTTCAGGAT3′ | 5′ACTTGCACGGGACATCTGT3′ | 1076 |
| 5′TGGGCTATAATCACTGCTCAC3′ | 5′GACCTGGCTCTACCATGAAAT3′ | 1127 |
| 5′CAACCTTCAGAATTTCCCCTA3′ | 5′AGGTTTGGCTATTTGGGACTA3′ | 1115 |
| 5′ATGTACCCACCGCCATACTA3′ | 5′GAGGTGACTTCCAGGTGATG3′ | 1037 |
| 5′AGCTAACAGCCCTGAAACATGA3′ | 5′GGTTCCGCATCCAACTTTATAT3′ | 1158 |
| 5′GGCTTCCGTATTCCTCAGTAG3′ | 5′CCATTGAAAGGAACTGGTGTA3′ | 1001 |
| 5′ATTGTGCATAGAGCCACGTA3′ | 5′AGTGGAAACCAAATGTGCTG3′ | 1152 |

**S1 Table. The sequence of *CTLA-4* primers in 5’ to 3’ direction**
